# Hydrogen Sulfide Signaling in Plants: Emerging Roles of Protein Persulfidation

**DOI:** 10.3389/fpls.2018.01369

**Published:** 2018-09-19

**Authors:** Angeles Aroca, Cecilia Gotor, Luis C. Romero

**Affiliations:** Institute of Plant Biochemistry and Photosynthesis, Consejo Superior de Investigaciones Científicas, Universidad de Sevilla, Seville, Spain

**Keywords:** Arabidopsis, cell signaling, cysteine, hydrogen sulfide, persulfidation, post-translational modification, proteomic

## Abstract

Hydrogen sulfide (H_2_S) has been largely referred as a toxic gas and environmental hazard, but recent years, it has emerged as an important gas-signaling molecule with effects on multiple physiological processes in both animal and plant systems. The regulatory functions of H_2_S in plants are involved in important processes such as the modulation of defense responses, plant growth and development, and the regulation of senescence and maturation. The main signaling pathway involving sulfide has been proven to be through protein persulfidation (alternatively called *S-*sulfhydration), in which the thiol group of cysteine (-SH) in proteins is modified into a persulfide group (-SSH). This modification may cause functional changes in protein activities, structures, and subcellular localizations of the target proteins. New shotgun proteomic approaches and bioinformatic analyses have revealed that persulfidated cysteines regulate important biological processes, highlighting their importance in cell signaling, since about one in 20 proteins in Arabidopsis is persulfidated. During oxidative stress, an increased persulfidation has been reported and speculated that persulfidation is the protective mechanism for protein oxidative damage. Nevertheless, cysteine residues are also oxidized to different post-translational modifications such *S*-nitrosylation or *S-*sulfenylation, which seems to be interconvertible. Thus, it must imply a tight cysteine redox regulation essential for cell survival. This review is aimed to focus on the current knowledge of protein persulfidation and addresses the regulation mechanisms that are disclosed based on the knowledge from other cysteine modifications.

## Introduction

Hydrogen sulfide (H_2_S) is an inorganic, flammable, water-soluble gas with a noticeable odor of rotten eggs. H_2_S has been historically considered as a pollutant and a toxic gas for life. Nevertheless, in the past decade, it has emerged as a new gaseous signaling molecule (gasotransmitter) in animal and plant cells and is as important as nitric oxide (NO), carbon monoxide (CO), and hydrogen peroxide (H_2_O_2_) (Garcia-Mata and Lamattina, 2010; Vandiver and Snyder, 2012; Kimura, 2014). Since 1996, when H_2_S was first described as an endogenous neuromodulator in animals (Abe and Kimura, 1996), an increasing number of articles have described its physiological effects on plants and animals. H_2_S is involved in many physiological and pathological processes in animals, such as apoptosis, inflammatory processes, the protective effects against hypoxia, neuromodulation, cell proliferation and cardioprotection, among others, as described in several recent reports and reviews (Wang, 2014; Olas, 2015; Paul and Snyder, 2015a).

In plants, the first descriptions of H_2_S effects are dated to the 1960s, when H_2_S was reported to influence the growth of vegetative plants and to affect disease resistance (Rodriguez-Kabana et al., 1965; Thompson and Kats, 1978). Mainly based on pharmacological approaches, H_2_S has been documented for its protective effects against different stresses, such as oxidative and metal stresses (Zhang et al., 2008; Wang et al., 2010; Zhang et al., 2010b; Li L. et al., 2012; Shen et al., 2013; Sun et al., 2013; Fang et al., 2016), drought and heat tolerance (Li Z.G. et al., 2012; Shen et al., 2013), and osmotic and saline stresses (Shi et al., 2013). H_2_S was also found to regulate important physiological processes in plants, such as stomatal closure/aperture (Garcia-Mata and Lamattina, 2010; Lisjak et al., 2010; Jin et al., 2013; Scuffi et al., 2014; Papanatsiou et al., 2015), the modulation of photosynthesis (Chen et al., 2011) and autophagy regulation (Álvarez et al., 2012a; Gotor et al., 2013; Romero et al., 2014; Laureano-Marin et al., 2016). However, an endogenous production of H_2_S was also observed in plants, and a possible cross-talk with NO was proposed due to the similarity of their physiological effects (Kolluru et al., 2015). All these observations have prompted interest in the scientific community to address the outstanding questions related to H_2_S signaling and its interaction with other gasotransmitters and hormones in plants.

This review is aimed to focus on the current knowledge of H_2_S as a signaling molecule, its mechanism of action and its role in plant physiology.

## Sulfide as an Emerging Signal Molecule in Plants

In plant systems, H_2_S production occurs mainly via the photosynthetic sulfate-assimilation pathway in chloroplasts in the reaction catalyzed by the sulfite reductase (SiR) (Takahashi et al., 2011; Garcia et al., 2015). During the synthesis of β-cyanoalanine, the enzyme cyanoalanine synthase c1 (CAS-C1) also generates sulfide in the mitochondria (Yamaguchi et al., 2000; Álvarez et al., 2012b). The enzyme L-cysteine desulfhydrase 1 (DES1) is the responsible of the major production of endogenous cytosolic H_2_S (Álvarez et al., 2010, 2012a; Gotor et al., 2010), nevertheless other enzymes have also been reported to produce sulfide, such as D-cysteine desulfhydrase (Riemenschneider et al., 2005) and Nifs-like proteins (ABA3) (Heidenreich et al., 2005). The sulfide concentration in chloroplasts is higher than that in the cytosol (125 and 55 μM, respectively) (Krueger et al., 2009), however, this sulfide is dissociated into its ionized forms due the basic pH inside organelles and therefore is unable to pass through the membranes to the cytosol (Kabil and Banerjee, 2010).

Hydrogen sulfide (H_2_S) is a particularly reactive molecule, and there is plenty of evidences that H_2_S interacts with other signaling molecules to modifying their signal. There is increasing interest in the interaction of sulfide with plant hormones such as abscisic acid (ABA) (Jin et al., 2013; Scuffi et al., 2014), giberellic acid (GA) (Xie et al., 2014), and ethylene (Liu et al., 2011; Liu et al., 2012). Further recent evidence suggests that H_2_S also plays a role in the hydrogen peroxide (H_2_O_2_) (Zhang et al., 2010a) and nitric oxide (NO) signaling pathways (Lisjak et al., 2011). Although numerous reports highlight the importance of H_2_S as a signaling molecule, its primary mechanism of action has been recently deciphered (Mustafa et al., 2009; Aroca et al., 2015). It has been explained through a new post-translational modification (PTM) of proteins, named persulfidation, where reactive cysteine residues on target proteins are modified via conversion of the thiol group (-SH) into a persulfide group (-SSH). This modification was inaccurately referred to as “*S-*sulfhydration”; however, since it does not imply any “hydration,” it was renamed as persulfidation (Filipovic, 2015). Persulfide adducts show an increased nucleophilicity compared to the thiol group and therefore, modified cysteines demonstrate a challenging greater reactivity (Paul and Snyder, 2012). That could be the reason persulfidation is widespread in nature and affects a higher percentage of proteins than reactive oxygen and nitrogen species (Mustafa et al., 2009; Ida et al., 2014).

The way H_2_S modifies specific targets is still unclear, since direct reaction of H_2_S and a thiol is thermodynamically unfavorable (Filipovic, 2015). Sulfane sulfur is a sulfur atom that has the unique ability to bind reversibly to other sulfur atoms to form hydropersulfides (R-S-SH) and polysulfides (-S-S_n_-S-). These polysulfides seems to be much more effective in persulfidation since they are more nucleophilic than H_2_S (Toohey, 1989, 2011). New low molecular weight (LMW) persulfides have recently emerged as potential mediators in sulfide signaling. In this regard, cysteine-persulfide (Cys-SSH), glutathione persulfide (GSSH) and its persulfurated species Cys-SS_n_H and GSS_n_H have been recognized as redox regulators (Kasamatsu et al., 2016; Kimura et al., 2017). Recently, the endogenous Cys-SSH production synthetized by prokaryotic and mammalian cysteinyl-tRNA synthetases (CARSs) using L-cysteine as substrate has been described. The cysteine polysulfides bound to tRNA are incorporated into polypeptides that are synthesized *de novo* in the ribosomes, suggesting that these enzymes are the principal cysteine persulfide synthases *in vivo* (Akaike et al., 2017).

Two extensive reviews have been recently published further explaining the chemical properties of persulfides and polysulfides (Cuevasanta et al., 2017; Filipovic et al., 2017). Due to the instability and high reactivity of persulfides, and its similarity to thiols, the efforts on developing detection methods have been a challenge.

The modified biotin switch assay (mBSM) was the first method used for a proteomic approach based on the analysis of persulfidated proteins in mammals (Mustafa et al., 2009). This method uses *S-*methyl-methanothiosulfonate (MMTS) to block free thiols. While the persulfide residues remain unreacted and are therefore available for subsequent reaction with the thiol-specific biotinylating agent biotin-HPDP. Lately, was used to detect for the first time the persulfidated proteins in plant systems (Aroca et al., 2015). A total of 106 persulfidated proteins were identified by liquid chromatography-mass spectrometry (LC-MS/MS) in Arabidopsis plants, which were mainly involved in photosynthesis, protein synthesis, and cell organization according to MapMan classification. Some of these identified proteins in plants were previously described to undergo persulfidation in mammals. Furthermore, the low concentration of sulfide produced an inactivation/activation effect on enzyme activities, which was reversible by reductants, demonstrating that sulfide had a biological role in plants through persulfidation, similar to mammalian systems.

Nevertheless, MMTS was questioned as a good blocking reagent since it could also react with persulfides and thus protein identification was understimated.

Several reactives have been recently reported and used in animal systems for the detection of persulfides. These are (i) the fluorescent Cy5-maleimide (Sen et al., 2012), (ii) maleimide-PEG_2_-biotin (Dóka et al., 2016), and (iii) iodoacetyl-PEG2-biotin (Gao et al., 2015); among others. However, most of the methods hitherto described have shown a weakness by lacking in specificity.

In plants, a new approach to detect persulfidated proteins was recently reported (Aroca et al., 2017a) based on the method previously described and named the tag-switch method (Zhang et al., 2014), which showed higher specificity than other methods described. This method employs methylsulfonylbenzothiazole (MSBT) to block both thiols and persulfide groups; then, a nucleophilic attack by the cyanoacetate-based reagent CN-biotin is performed labeling only the persulfide groups, which are purified with streptavidin conjugates and analyzed by Western blot, or directly by LC-MS/MS. This study revealed that 2,015 proteins (5% of the Arabidopsis proteome) were modified by persulfidation and that approximately 3,200 proteins were potentially targets for this modification in mature plants. This new method increased the number of persulfidated targets in plants from 106 to more than 2,000 (Aroca et al., 2015, 2017a). These proteins were involved in the regulation of important biochemical pathways for cell survival. However, these data were obtained from plants grown under physiological conditions. Therefore, the number of persulfidated proteins may be higher under stress conditions where sulfide plays a signaling role.

## Biological Importance of Persulfidation in Plants

Although a high number of persulfidated proteins have been identified in both plant and animal systems, the functional impact of this modification in cells is starting to be clarified. Proteins modified by persulfidation show functional changes in enzymatic activities, structures and in subcellular localizations (Mustafa et al., 2009; Aroca et al., 2015, 2017b; Kimura, 2015; Paul and Snyder, 2015b). The biological importance of this protein modification in plant systems was initially demonstrated by the enzymatic activity of chloroplastic glutamine synthetase (GS2), cytosolic ascorbate peroxidase (APX1), and cytosolic glyceraldehyde 3-phosphate dehydrogenase (GapC1) (Aroca et al., 2015). The effect of persulfidation of these enzymes showed activity activation for APX1 and GapC1, while decreased activity was found for GS2; this effect was reversible by reducing agents.

The GO categorization of persulfidated proteins identified in Arabidopsis by a shot-gun proteomic assay revealed that persulfidation is involved in the regulation of important biological processes, such as carbon metabolism, plant responses to abiotic stresses, plant growth and development, and RNA translation (Aroca et al., 2017a). Moreover, the cytosolic or nuclear localization of GapC1 is regulated by persulfidation, and the persulfidated cysteine residue has been identified in nuclear fraction (Aroca et al., 2017b). In addition to the metabolic functions, GapC plays roles in mRNA regulation, transcriptional activation and apoptosis depending on its nuclear translocation outcome (Ortiz-Ortiz et al., 2010). Thus, persulfidation plays a key role not only in regulating the activity of modified proteins; it also may regulate cellular localization of proteins with significant consequences in plant systems.

The role of sulfide in another important biological process such as autophagy has also been described (Álvarez et al., 2012a; Laureano-Marin et al., 2016). Sulfide is able to inhibit the autophagy induced in Arabidopsis roots under nutrient deprivation; this repression is a mechanism independent of redox conditions. As described, H_2_S inhibits autophagy by preventing the ATG8 (autophagy-related ubiquitin-like protein) accumulation (Álvarez et al., 2012a). The mechanism of this inhibition by sulfide is still unclear, but Gotor and colleagues have speculated that this regulation might be through persulfidation of the enzymes involved in the autophagosome formation (Gotor et al., 2013). Furthermore, the ubiquitin-like systems ATG7-ATG10 and ATG7-ATG3, and the cysteine protease ATG4, which are essential proteins involved in autophagy, sense cellular redox alterations by their reactive Cys residues (Filomeni et al., 2010). Recently, a high throughput proteomic approach in Arabidopsis leaves revealed the susceptibility to be persulfidated of some autophagy (ATG)-related proteins, ATG18a, ATG3, ATG5, and ATG7 (Aroca et al., 2017a). In yeast and algae, ATG4 is regulated by thioredoxin, suggesting the involvement of a redox PTM in the regulation of ATG4 activity (Perez-Perez et al., 2014). In mammals, several PTMs play important roles in the regulation of autophagy. ATG4b and ATG1 are regulated by phosphorylation and *S-*nitrosylation (Li et al., 2017; Pengo et al., 2017; Sanchez-Wandelmer et al., 2017), and Caspase-3, which is also essential for autophagic activity, is persulfidated at Cys^163^ associating this modification to the cytoprotective effect of sulfide (Marutani et al., 2015).

The results in Arabidopsis suggest that persulfidation may be the molecular mechanism through which sulfide regulates autophagy in plant cells. However, the identification of other targets involved in autophagy requires further specific studies, and the functional role of persulfidation in autophagy has not been sufficiently studied.

**FIGURE 1:**
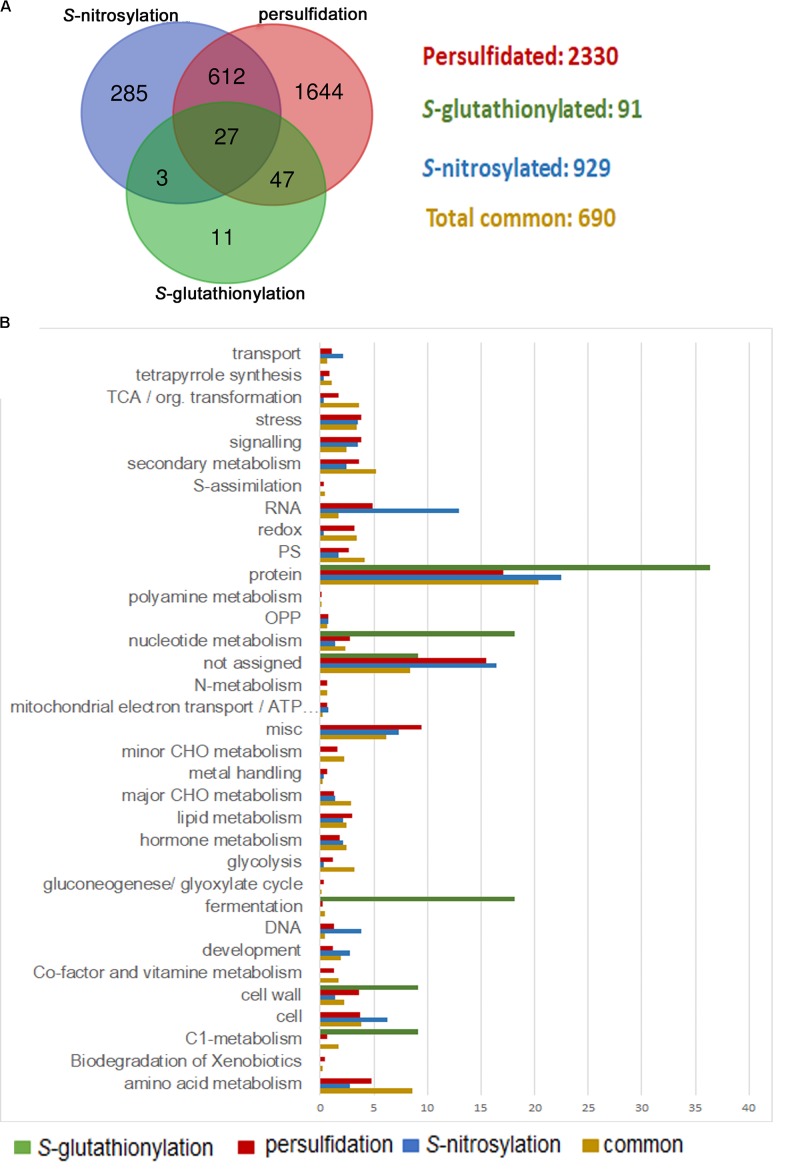
Comparison of persulfidated, *S-*nitrosylated, and *S-*glutathionylated proteins identified in Arabidopsis plants. **(A)** Venn diagram of total persulfidated (Aroca et al., 2017a), *S-*nitrosylated (Hu et al., 2015), and *S-*glutathionylated (Lee et al., 2004; Dixon et al., 2005; Lindermayr et al., 2010; Palmieri et al., 2010; Bedhomme et al., 2012) identified proteins in Arabidopsis plants. **(B)** Functional classification of gene ontology (GO) terms categorized by biological processes.

Many other biological functions must be precisely regulated through sulfide and the crosstalk with other signaling molecules, including H_2_O_2_ and NO, and other antioxidant molecules such as glutathione, which has been integrated in redox signaling. In this sense, protein thiols are crucial players since they can be modified by different PTMs, showing an opposite regulation in some cases. H_2_O_2_ directly attacks the catalytic cysteine of GAPC, causing a strong inhibition of the enzyme activity (Hancock et al., 2005). *S-*nitrosylation of GAPC also abolishes its catalytic activity, whereas persulfidation increases its activity (Lindermayr et al., 2005; Aroca et al., 2015). Besides, this enzyme may also undergo *S-*glutathionylation, which also inhibits its activity (Zaffagnini et al., 2007; Bedhomme et al., 2012). Likewise, oxidation of glutamine synthetase by H_2_O_2_ inhibits its activity (Ortega et al., 1999) in a similar manner as *S-*nitrosylation and persulfidation (Melo et al., 2011; Aroca et al., 2015). Moreover, *S-*glutathionylation of glutamine synthetase can also regulate its activity (Dixon et al., 2005). Aroca et al. (2017a) showed that 639 proteins were targets for both *S-*nitrosylation and persulfidation. Although just a few proteins (91) have been identified in Arabidopsis as *S-*glutathionylated proteins in the literature (Lee et al., 2004; Dixon et al., 2005; Leferink et al., 2009; Lindermayr et al., 2010; Palmieri et al., 2010; Bedhomme et al., 2012), 85% of them may also undergo *S-*nitrosylation or persulfidation (**Figure 1A**). A large amount of the proteins modified by these three PTMs are common (a total of 690), meaning they can be modified by at least two of these PTMs. Functional classification of the GO terms categorized by biological processes shows that these common proteins are mainly involved in protein metabolism (20%). However, they are even more represented in amino acid metabolism (8.5%), glycolysis (3.1%), tricarboxylic acid cycle (3.6%), redox regulation (3.4%), and secondary metabolism (5.2%) in comparison with those proteins that are modified by just one of these PTMs (**Figure 1B**). Thus, these pathways must be finely regulated by different PTMs in a cysteine residue, suggesting a crosstalk among these signaling molecules under different stress conditions.

Arabidopsis plants exogenously treated with H_2_S also showed changes in the transcriptome (Huang et al., 2002; Álvarez et al., 2012a). Based on proteomic approaches, several transcription factors (TFs) and chromatin modifiers (CMs) such as histones, acetyltransferases, and methyltransferases, have been identified as targets for persulfidation (Sen et al., 2012; Aroca et al., 2017a). This modification affects their specificity to DNA and their binding affinity resulting in distinct transcriptional responses. In addition, this PTM of nuclear proteins can modulate transcription by affecting their subcellular localization or by regulating the association with their binding partners (Filipovic, 2015; Aroca et al., 2017b). Thus, sulfide is also involved in epigenetic regulation of chromatin by histone modification and chromatin structure alteration (Kamat et al., 2015; Yang, 2015), highlighting an interesting signaling role of sulfide through persulfidation that could also be functioning in plants.

Plants are exposed to several stresses, which cause oxidative stress due to the accumulation of reactive oxygen species (ROS) and nitrogen species (RNS) (Huang et al., 2012; Schieber and Chandel, 2014). Under these stress conditions, cysteine thiols may undergo different PTMs such as *S-*nitrosylation (SNO), *S*-glutathionylation (SSG), and *S*-sulfenylation (SOH). However, these oxidized compounds can be reduced in the cells by reducing agents, such as glutathione (GSH), thioredoxin (Trx), and glutaredoxin (Grx) (Sevilla et al., 2015). Nevertheless, when the stress remains, irreversible modifications of thiols occurs, such as sulfinic (RSO_2_H), and sulfonic acids (RSO_3_H). In that sense, persulfidation (SSH) is believed to account for the protective effect against ROS/RNS, since persulfidated proteins will react with ROS/RNS and form an adduct (RSSO3H) that may be restored by thioredoxin to free thiol (Wedmann et al., 2016; Filipovic and Jovanović, 2017). Most antioxidant enzymes are finely regulated by different PTMs under specific environmental stress conditions. In that sense, APX1 is inactivated by the oxidation of Cys^32^, while glutathionylation protects the enzyme from irreversible oxidation (Kitajima et al., 2008). The same site Cys^32^ can also be *S-*nitrosylated by NO and persulfidated by hydrogen sulfide, which increases the activity of the enzyme (Begara-Morales et al., 2014; Aroca et al., 2015). The fact that APX1 is modified by different PTMs means that this enzyme must be finely regulated under specific stress conditions.

Nitric Oxide increases the activities of antioxidant enzymes such as catalase (CAT), superoxide dismutase (SOD), ascorbate peroxidase (APX), glutathione reductase (GPX), and peroxidase (POD); therefore, NO may stimulate the antioxidant system to decrease oxidative stress (Lamattina et al., 2003; Laspina et al., 2005). NO levels increase in plants under drought stress, which helps plants to mitigate the negative effects of water deficit (Shi et al., 2014), and NO is an important player in ABA-induced stomatal closure, minimizing plant transpiration (Seabra and Oliveira, 2016). Exogenous H_2_S is found to induce stomatal closure through the regulation of ATP-binding cassette (ABC) transporters, while scavenging H_2_S can partially block ABA-dependent stomatal closure, indicating the protective role of H_2_S in plants against drought stress (Garcia-Mata and Lamattina, 2010). Sulfide-induced stomatal closure can be reverted by cPTIO (a NO-specific scavenger), also confirming that the function of sulfide in stomatal closure is mediated by NO. Furthermore, several proteins that play essential roles in the ABA-dependent regulation of stomatal movement, are modified by persulfidation and *S-*nitrosylation (Wang et al., 2015; Aroca et al., 2017a). These findings suggest a crosstalk between NO and H_2_S in drought stress mediated by *S-*nitrosylation and persulfidation, respectively.

## Concluding Remarks

An increasing number of articles regarding persulfidation have been published in the last years on different organisms, and many proteins have been found to be modified by sulfide through persulfidation. Nevertheless, the functional role of this modification in specific targets must be studied to validate these data. Furthermore, the specificity on cysteine residues is still unclear since, in some targets, the modified cysteine is the active site, but not in others. Additionally, the extent of the interaction between several signaling molecules into the same cysteine residue deserves more investigation. Understanding the interplay among them will enlarge our knowledge on the biochemical cascade triggered in plant cells against different stresses.

A large number of laboratory experiments have revealed the protective effect of sulfide in plants to overcome different environmental stresses. A better understanding of persulfidation in these stress events would help to address biotechnological applications in order to improve the productivity of crops and protection against abiotic stress processes.

## Author Contributions

AA organized and prepared all this manuscript. CG and LR contributed for writing and reviewing the major part of the manuscript.

## Conflict of Interest Statement

The authors declare that the research was conducted in the absence of any commercial or financial relationships that could be construed as a potential conflict of interest.
